# Supplementary figures and images for: Roles of the membrane-reentrant β-hairpin-like loop of RseP protease in selective substrate cleavage
Source: eLife. 2015 Oct 8;4:e08928. doi: 10.7554/eLife.08928 (PMC4597795; doi:10.7554/eLife.08928)

Figure 2—source data

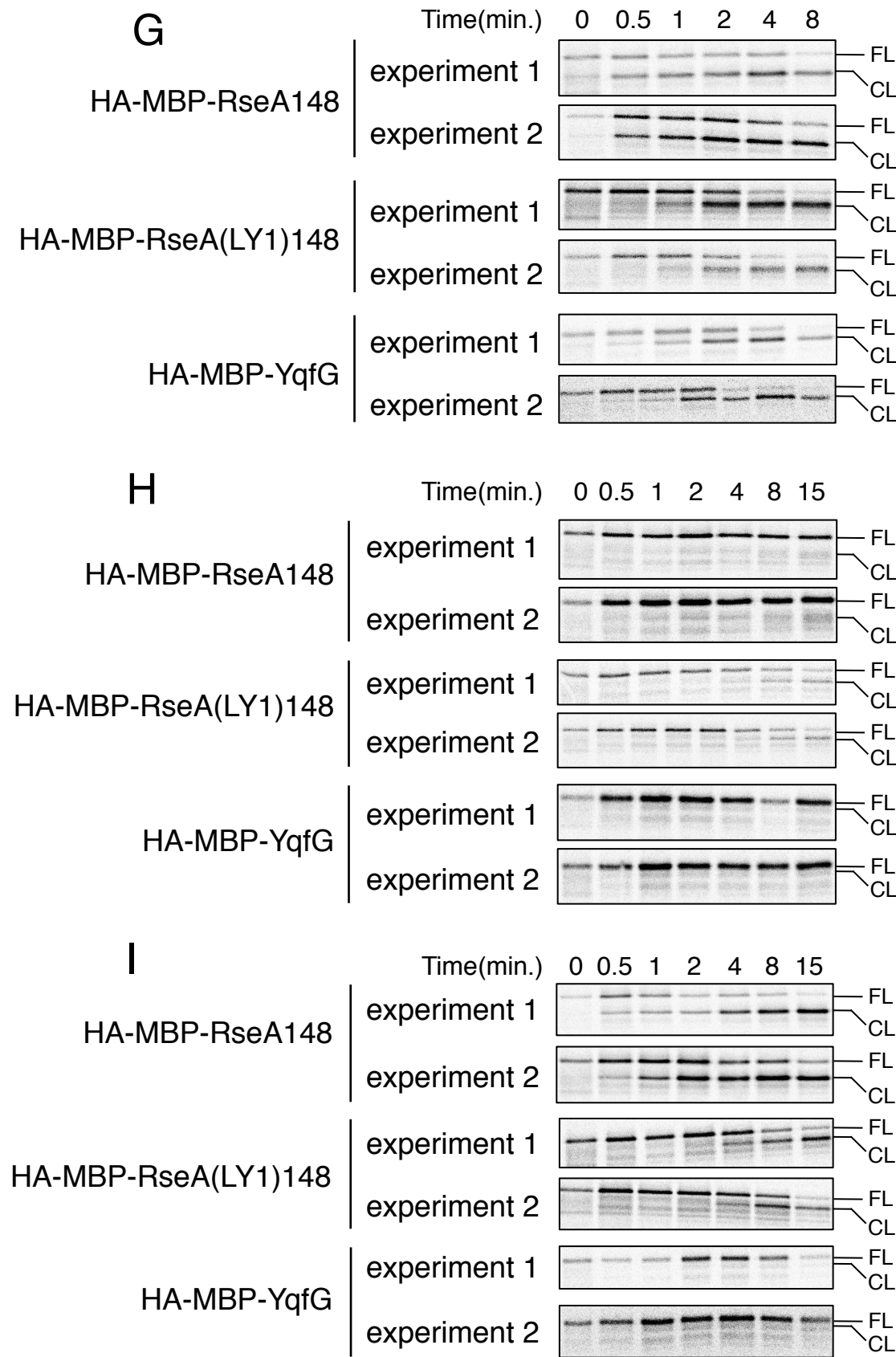

Supplement: Figure 2—source data 1. — DOI: http://dx.doi.org/10.7554/eLife.08928.005 [file elife08928s001.zip › Figure 2 source data 1/Figure 2G-I gel images.pdf]

Figure 6—source data

D

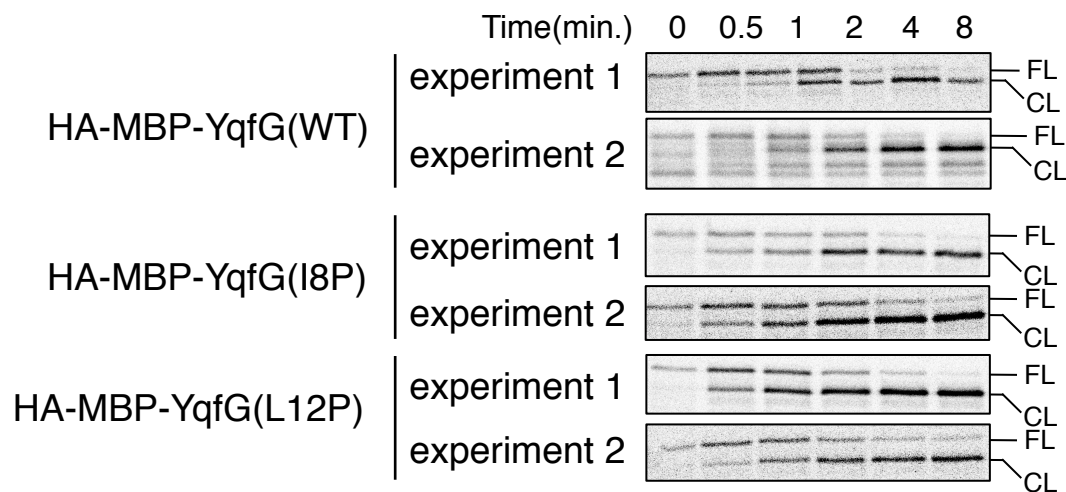

E

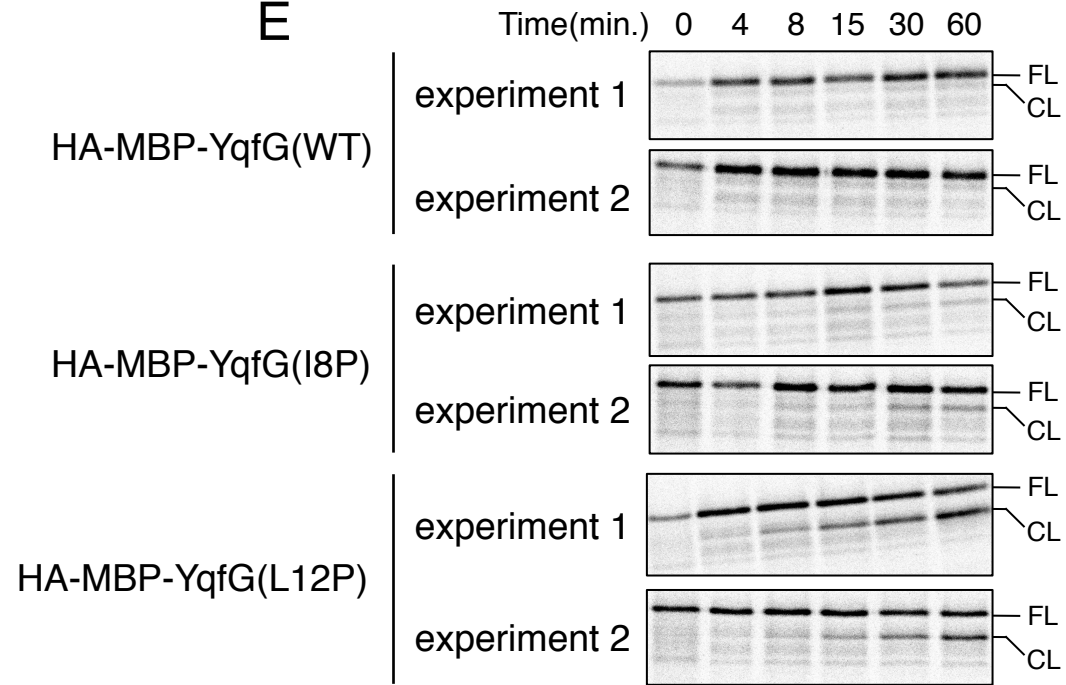

F

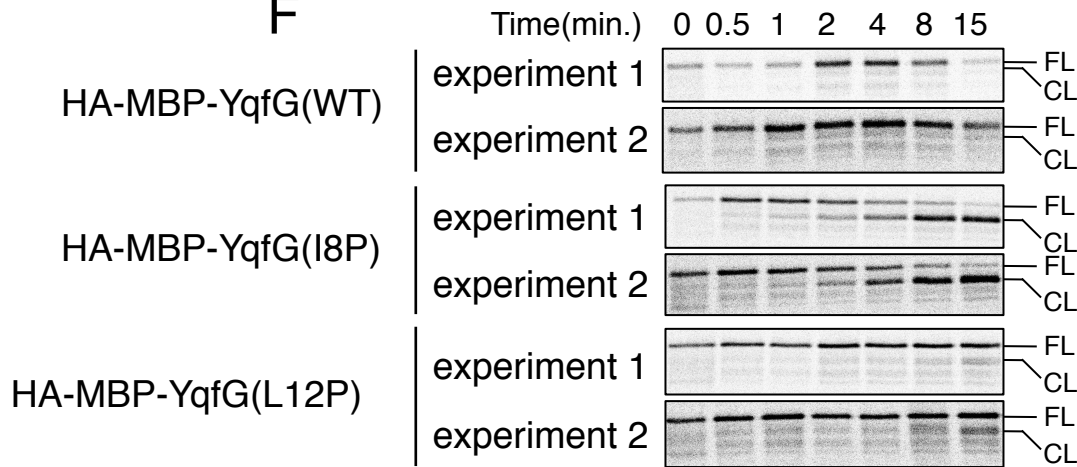

Supplement: Figure 6—source data 1. — DOI: http://dx.doi.org/10.7554/eLife.08928.014 [file elife08928s002.zip › Figure 6 source data 1/Figure 6D-F gel images.pdf]
