# Supplementary material for: Roles of the membrane-reentrant β-hairpin-like loop of RseP protease in selective substrate cleavage
Source: eLife. 2015 Oct 8;4:e08928. doi: 10.7554/eLife.08928 (PMC4597795; doi:10.7554/eLife.08928)
Supplement: Figure 6—source data 1. — DOI: http://dx.doi.org/10.7554/eLife.08928.014 [file elife08928s002.zip › Figure 6 source data 1/Figure 6D-F source data explanation.docx]

**Figure 6 source data 1. Gel images and quantified band intensity data for the pulse-chase experiments for Figure 6D-F.**

KA306 cells harbouring an appropriate combination of plasmids encoding the indicated RseP-HM mutants and HA-MBP-YqfG mutants were grown in M9-based medium containing 1 mM IPTG at 30°C. The cells were labelled with [^35^S]-methionine for 30 s and were chased with unlabelled methionine for the indicated periods. Proteins were immunoprecipitated using agarose-conjugated anti-HA antibody and were analysed by 10% Laemmli–SDS-PAGE. The results of the two independent experiments are shown. Intensities of FL (full-length form) and CL (cleaved form) bands were quantitated and cleavage (%) was calculated using the following equation: cleavage (%) = 100 × (CL)/[(FL) + (CL)] after correction of Met contents.
