# Supplementary material for: Roles of the membrane-reentrant β-hairpin-like loop of RseP protease in selective substrate cleavage
Source: eLife. 2015 Oct 8;4:e08928. doi: 10.7554/eLife.08928 (PMC4597795; doi:10.7554/eLife.08928)
Supplement: Supplementary file 1. — Table S1. Strains used in this study. DOI: http://dx.doi.org/10.7554/eLife.08928.017 [file elife08928s003.docx]

Supplementary file 1

Table S1. Strains used in this study

| Strains | Genotype | References |
| --- | --- | --- |
| AD16 | Δ(*pro*-*lac*) *thi* /F’*lacI^q^ Z*M15 *Y*^+^ *pro*^+^ | (*Akiyama and Ito, 1985*) |
| AD1811 | AD16 Δ*rseA*::*cat* | (*Kanehara et al, 2002*) |
| AD1839 | AD16 Δ*rseA*::*cat* Δ*degS*::*tet* | (*Kanehara et al, 2002*) |
| AD1840 | AD16 Δ*rseA*::*cat* Δ*rseP*::*kan* Δ*degS*::*tet* | (*Kanehara et al, 2002*) |
| AD2328 | AD16 Δ*rseA* Δ*rseP*::*kan* | (*Saito et al, 2011*) |
| CU141 | MC4100 /F’*lac^+^lacI^q^* | (*Akiyama et al, 1994*) |
| CAG16037 | MC1061 φλ[*rpoHP3*::*lacZ*] | (*Mecsas et al, 1993*) |
| JW0940 | Keio Collection | (*Baba et al, 2006*) |
| JW2203 | Keio Collection | (*Baba et al, 2006*) |
| KA306 | AD16 Δ*rseA* Δ*rseP*::*kan* Δ*clpP*::*cat* | This study |
| KA363 | MC4100 Δ*ompA* Δ*ompC* Δ*rseP*::*kan* | This study |
| KA418 | MC4100 Δ*ompA* Δ*ompC* Δ*rseP*::*kan* /F’*lac^+^lacI^q^* | This study |
| KA438 | MC4100 Δ*ompA* Δ*ompC* Δ*rseP*::*kan* Δ*degS*::*tet* /F’*lac^+^lacI^q^* | This study |
| KK31 | AD16 Δ*rseP*::*kan* Δ(*srl*-*recA*)*306*::Tn*10* /pKK6(P*ara*-*rseP*) | (*Kanehara et al, 2001*) |
| KK211 | AD16 Δ*rseA*::*cat* Δ*rseP*::*kan* | (*Kanehara et al, 2002*) |
| KK374 | CU141 Δ*rseA*::*cat* Δ*rseP*::*kan* Δ*degS*::*tet* | (*Akiyama et al, 2004*) |
| MC4100 | *araD*139 Δ(*argF*-*lac*)*U*169 *rpsL*150 *relA*1 *flbB*5301 | (*Silhavy et al, 1984*) |
| YH426 | MC4100 Δ*ompA* Δ*ompC* | This study |

Akiyama Y, Ito K. 1985. The SecY membrane component of the bacterial protein export machinery: analysis by new electrophoretic methods for integral membrane proteins. *EMBO J* **4:** 3351-3356.

Akiyama Y, Kanehara K, Ito K. 2004. RseP (YaeL), an *Escherichia coli* RIP protease, cleaves transmembrane sequences. *EMBO J* **23:** 4434-4442. doi: 10.1038/sj.emboj.7600449

Akiyama Y, Ogura T, Ito K. 1994. Involvement of FtsH in protein assembly into and through the membrane. I. Mutations that reduce retention efficiency of a cytoplasmic reporter. *J Biol Chem* **269:** 5218-5224.

Baba T, Ara T, Hasegawa M, Takai Y, Okumura Y, Baba M, Datsenko KA, Tomita M, Wanner BL, Mori H. 2006. Construction of *Escherichia coli* K-12 in-frame, single-gene knockout mutants: the Keio collection. *Mol Syst Biol* **2:** 2006.0008. doi: 10.1038/msb4100050

Kanehara K, Akiyama Y, Ito K. 2001. Characterization of the *yaeL* gene product and its S2P-protease motifs in *Escherichia coli*. *Gene* **281:** 71-79. doi: 10.1016/S0378-1119(01)00823-X

Kanehara K, Ito K, Akiyama Y. 2002. YaeL (EcfE) activates the σ^E^ pathway of stress response through a site-2 cleavage of anti-σ^E^, RseA. *Genes Dev* **16:** 2147-2155. doi: 10.1101/gad.1002302

Mecsas J, Rouviere PE, Erickson JW, Donohue TJ, Gross CA. 1993. The activity of σ^E^, an *Escherichia coli* heat-inducible sigma-factor, is modulated by expression of outer membrane proteins. *Genes Dev* **7:** 2618-2628. doi: 10.1128/JB.185.8.2512-2519.2003

Saito A, Hizukuri Y, Matsuo E, Chiba S, Mori H, Nishimura O, Ito K, Akiyama Y. 2011. Post-liberation cleavage of signal peptides is catalyzed by the site-2 protease (S2P) in bacteria. *Proc Natl Acad Sci USA* **108:** 13740-13745. doi: 10.1073/pnas.1108376108

Silhavy TJ, Berman ML, Enquist LW. 1984. Experiments with Gene Fusions, Cold Spring Harbor, NewYork: Cold Spring Harbor Laboratory Press.
