# Supplementary material for: Roles of the membrane-reentrant β-hairpin-like loop of RseP protease in selective substrate cleavage
Source: eLife. 2015 Oct 8;4:e08928. doi: 10.7554/eLife.08928 (PMC4597795; doi:10.7554/eLife.08928)
Supplement: Supplementary file 2. — Table S2. Plasmids used in this study. DOI: http://dx.doi.org/10.7554/eLife.08928.018 [file elife08928s004.doc]

Supplementary file 2

Table S2. Plasmids used in this study

| Plasmids | Vector | Encoded proteins or descriptions | References or sources |
| --- | --- | --- | --- |
| pBAD33 |  | Expression vector; P*_ara_*, Cm^R^ | *(Guzman et al, 19*95) |
| pSTD689 |  | Expression vector; P*_lac_*, Spc^R^ | *(Kanehara et al, 20*03) |
| pTWV228 |  | Expression vector; P*_lac_*, Amp^R^ | Takara Bio |
| pTYE007 |  | Expression vector; P*_lac_*, Amp^R^ | *(Akiyama et al, 19*95) |
| pUC118 |  | Expression vector; P*_lac_*, Amp^R^ | Takara Bio |
| pCP20 |  | pSC101-derivative encoding Flp recombinase Amp^R^, Cm^R^ | *(Cherepanov and Wackernagel, 19*95) |
| pEVOL  -pBpF |  | p15A-derivative encoding mutant *M. jannaschii* aminoacyl-tRNA synthetase for *p*BPA and the corresponding suppressor tRNA; Cm^R^ | *(Young et al, 20*10) |
| pKA1 | pUC118 | RseP(Δloop)-His_6_-Myc | This study |
| pKA19 | pTYE007 | RseP(E23Q)-His_6_-Myc | This study |
| pKA22 | pTYE007 | RseP(I61C)-His_6_-Myc | This study |
| pKA23 | pTYE007 | RseP(A62C)-His_6_-Myc | This study |
| pKA24 | pTYE007 | RseP(L63C)-His_6_-Myc | This study |
| pKA25 | pTYE007 | RseP(I64C)-His_6_-Myc | This study |
| pKA26 | pTYE007 | RseP(P65C)-His_6_-Myc | This study |
| pKA27 | pTYE007 | RseP(L66C)-His_6_-Myc | This study |
| pKA28 | pTYE007 | RseP(G67C)-His_6_-Myc | This study |
| pKA29 | pTYE007 | RseP(G68C)-His_6_-Myc | This study |
| pKA30 | pTYE007 | RseP(Y69C)-His_6_-Myc | This study |
| pKA31 | pTYE007 | RseP(V70C)-His_6_-Myc | This study |
| pKA32 | pTYE007 | RseP(K71C)-His_6_-Myc | This study |
| pKA33 | pTYE007 | RseP(M72C)-His_6_-Myc | This study |
| pKA34 | pTYE007 | RseP(L73C)-His_6_-Myc | This study |
| pKA35 | pTYE007 | RseP(D74C)-His_6_-Myc | This study |
| pKA36 | pTYE007 | RseP(E75C)-His_6_-Myc | This study |
| pKA37 | pTYE007 | RseP(E23Q/I61C)-His_6_-Myc | This study |
| pKA38 | pTYE007 | RseP(E23Q/A62C)-His_6_-Myc | This study |
| pKA39 | pTYE007 | RseP(E23Q/L63C)-His_6_-Myc | This study |
| pKA40 | pTYE007 | RseP(E23Q/I64C)-His_6_-Myc | This study |
| pKA41 | pTYE007 | RseP(E23Q/P65C)-His_6_-Myc | This study |
| pKA42 | pTYE007 | RseP(E23Q/L66C)-His_6_-Myc | This study |
| pKA43 | pTYE007 | RseP(E23Q/G67C)-His_6_-Myc | This study |
| pKA44 | pTYE007 | RseP(E23Q/G68C)-His_6_-Myc | This study |
| pKA45 | pTYE007 | RseP(E23Q/Y69C)-His_6_-Myc | This study |
| pKA46 | pTYE007 | RseP(E23Q/V70C)-His_6_-Myc | This study |
| pKA47 | pTYE007 | RseP(E23Q/K71C)-His_6_-Myc | This study |
| pKA48 | pTYE007 | RseP(E23Q/M72C)-His_6_-Myc | This study |
| pKA49 | pTYE007 | RseP(E23Q/L73C)-His_6_-Myc | This study |
| pKA50 | pTYE007 | RseP(E23Q/D74C)-His_6_-Myc | This study |
| pKA51 | pTYE007 | RseP(E23Q/E75C)-His_6_-Myc | This study |
| pKA52 | pUC118 | RseP(E23Q)-His_6_-Myc | This study |
| pKA53 | pUC118 | RseP(E23Q/Δloop)-His_6_-Myc | This study |
| pKA55 | pUC118 | RseP(I61P)-His_6_-Myc | This study |
| pKA56 | pUC118 | RseP(A62P)-His_6_-Myc | This study |
| pKA57 | pUC118 | RseP(L63P)-His_6_-Myc | This study |
| pKA58 | pUC118 | RseP(I64P)-His_6_-Myc | This study |
| pKA59 | pUC118 | RseP(G68P)-His_6_-Myc | This study |
| pKA60 | pUC118 | RseP(V70P)-His_6_-Myc | This study |
| pKA64 | pUC118 | RseP(E23Q/N389L)-His_6_-Myc | This study |
| pKA65 | pSTD689 | HA-MBP-RseA148 | This study |
| pKA67 | pUC118 | RseP(L66P)-His_6_-Myc | This study |
| pKA68 | pUC118 | RseP(G67P)-His_6_-Myc | This study |
| pKA69 | pUC118 | RseP(Y69P)-His_6_-Myc | This study |
| pKA70 | pUC118 | RseP(K71P)-His_6_-Myc | This study |
| pKA72 | pUC118 | RseP(E75P)-His_6_-Myc | This study |
| pKA73 | pUC118 | RseP(E23Q/I61*amber*)-His_6_-Myc | This study |
| pKA74 | pUC118 | RseP(E23Q/A62*amber*)-His_6_-Myc | This study |
| pKA75 | pUC118 | RseP(E23Q/L63*amber*)-His_6_-Myc | This study |
| pKA76 | pUC118 | RseP(E23Q/I64*amber*)-His_6_-Myc | This study |
| pKA77 | pUC118 | RseP(E23Q/P65*amber*)-His_6_-Myc | This study |
| pKA78 | pUC118 | RseP(E23Q/L66*amber*)-His_6_-Myc | This study |
| pKA79 | pUC118 | RseP(E23Q/G67*amber*)-His_6_-Myc | This study |
| pKA80 | pUC118 | RseP(E23Q/G68*amber*)-His_6_-Myc | This study |
| pKA81 | pUC118 | RseP(E23Q/Y69*amber*)-His_6_-Myc | This study |
| pKA82 | pUC118 | RseP(E23Q/V70*amber*)-His_6_-Myc | This study |
| pKA83 | pUC118 | RseP(E23Q/K71*amber*)-His_6_-Myc | This study |
| pKA84 | pUC118 | RseP(E23Q/M72*amber*)-His_6_-Myc | This study |
| pKA85 | pUC118 | RseP(E23Q/L73*amber*)-His_6_-Myc | This study |
| pKA86 | pUC118 | RseP(E23Q/D74*amber*)-His_6_-Myc | This study |
| pKA87 | pUC118 | RseP(E23Q/E75*amber*)-His_6_-Myc | This study |
| pKA94 | pUC118 | RseP(D74P)-His_6_-Myc | This study |
| pKA99 | pUC118 | RseP(I61*amber*)-His_6_-Myc | This study |
| pKA100 | pUC118 | RseP(A62*amber*)-His_6_-Myc | This study |
| pKA101 | pUC118 | RseP(L63*amber*)-His_6_-Myc | This study |
| pKA102 | pUC118 | RseP(I64*amber*)-His_6_-Myc | This study |
| pKA103 | pUC118 | RseP(P65*amber*)-His_6_-Myc | This study |
| pKA104 | pUC118 | RseP(L66*amber*)-His_6_-Myc | This study |
| pKA105 | pUC118 | RseP(G67*amber*)-His_6_-Myc | This study |
| pKA106 | pUC118 | RseP(G68*amber*)-His_6_-Myc | This study |
| pKA107 | pUC118 | RseP(Y69*amber*)-His_6_-Myc | This study |
| pKA108 | pUC118 | RseP(V70*amber*)-His_6_-Myc | This study |
| pKA109 | pUC118 | RseP(K71*amber*)-His_6_-Myc | This study |
| pKA110 | pUC118 | RseP(M72*amber*)-His_6_-Myc | This study |
| pKA111 | pUC118 | RseP(L73*amber*)-His_6_-Myc | This study |
| pKA112 | pUC118 | RseP(D74*amber*)-His_6_-Myc | This study |
| pKA113 | pUC118 | RseP(E75*amber*)-His_6_-Myc | This study |
| pKA117 | pTYE007 | RseP(Cys-less, Δloop)-His_6_-Myc | This study |
| pKA118 | pTYE007 | RseP(Cys-less, Δloop/V13C)-His_6_-Myc | This study |
| pKA119 | pTYE007 | RseP(Cys-less, Δloop/I386C)-His_6_-Myc | This study |
| pKA120 | pTYE007 | RseP(Cys-less, Δloop/A77C)-His_6_-Myc | This study |
| pKA121 | pTYE007 | RseP(Cys-less, Δloop/A136C)-His_6_-Myc | This study |
| pKA122 | pUC118 | RseP(E23Q/G67P)-His_6_-Myc | This study |
| pKA123 | pUC118 | RseP(E23Q/G68P)-His_6_-Myc | This study |
| pKA124 | pUC118 | RseP(E23Q/Y69P)-His_6_-Myc | This study |
| pKA125 | pUC118 | RseP(E23Q/V70P)-His_6_-Myc | This study |
| pKA126 | pUC118 | RseP(E23Q/K71P)-His_6_-Myc | This study |
| pKA127 | pUC118 | RseP(E23Q/D74P)-His_6_-Myc | This study |
| pKA143 | pUC118 | RseP(E23Q/Y69*amber*/N389L)-His_6_-Myc | This study |
| pKA146 | pUC118 | RseP(E23Q/K71*amber*/N389L)-His_6_-Myc | This study |
| pKA147 | pUC118 | RseP(E23Q/D74*amber*/N389L)-His_6_-Myc | This study |
| pKA148 | pUC118 | RseP(M72P)-His_6_-Myc | This study |
| pKA149 | pUC118 | RseP(L73P)-His_6_-Myc | This study |
| pKA150 | pUC118 | RseP(E23Q/I61P)-His_6_-Myc | This study |
| pKA151 | pUC118 | RseP(E23Q/A62P)-His_6_-Myc | This study |
| pKA152 | pUC118 | RseP(E23Q/L63P)-His_6_-Myc | This study |
| pKA153 | pUC118 | RseP(E23Q/I64P)-His_6_-Myc | This study |
| pKA154 | pTYE007 | RseP(A62C/G68P)-His_6_-Myc | This study |
| pKA195 | pSTD689 | HA-RseA148 | This study |
| pKA210 | pSTD689 | HA-MBP-YoaJ | This study |
| pKA268 | pUC118 | HA-MBP-YoaJ | This study |
| pKA283 | pSTD689 | HA-MBP-RseA(LY1-F20P)148 | This study |
| pKA284 | pSTD689 | HA-MBP-RseA(LY1-F21P)148 | This study |
| pKD5 | pTYE007 | RseP(V13C)-His_6_-Myc | *(Koide et al, 20*07) |
| pKD11 | pTYE007 | RseP(I386C)-His_6_-Myc | *(Koide et al, 20*07) |
| pKD15 | pTYE007 | RseP(A136C)-His_6_-Myc | *(Koide et al, 20*07) |
| pKD35 | pTYE007 | RseP(A77C)-His_6_-Myc | *(Koide et al, 20*07) |
| pKD49 | pTYE007 | RseP(H22C)-His_6_-Myc | *(Koide et al, 20*07) |
| pKD50 | pTYE007 | RseP(H23C)-His_6_-Myc | *(Koide et al, 20*07) |
| pKD99 | pSTD689 | HA-RseA140 | *(Koide et al, 20*08) |
| pKD124 | pSTD689 | HA-RseA(A108C)140 | *(Koide et al, 20*08) |
| pKD126 | pSTD689 | HA-RseA(Cys-less)140 | *(Koide et al, 20*08) |
| pKH441 | pTWV228 | HflD | *(Kihara et al, 20*01) |
| pKK6 | pBAD33 | RseP | *(Kanehara et al, 20*01) |
| pKK34 | pTWV228 | RseP(E23Q)-His_6_-Myc | *(Kanehara et al, 20*01) |
| pKK49 | pUC118 | RseP-His_6_-Myc | *(Akiyama et al, 20*04) |
| pMZ14 | pSTD689 | HA-MBP-YqfG | This study |
| pMZ65 | pSTD689 | HA-MBP-YqfG(L12P) | This study |
| pMZ77 | pSTD689 | HA-MBP-YqfG(I8P) | This study |
| pMZ78 | pSTD689 | HA-MBP-YqfG(F9P) | This study |
| pMZ79 | pSTD689 | HA-MBP-YqfG(S10P) | This study |
| pMZ80 | pSTD689 | HA-MBP-YqfG(L11P) | This study |
| pMZ81 | pSTD689 | HA-MBP-YqfG(L13P) | This study |
| pMZ82 | pSTD689 | HA-MBP-YqfG(F15P) | This study |
| pMZ92 | pSTD689 | HA-MBP-YqfG(L12N) | This study |
| pMZ94 | pSTD689 | HA-MBP-YqfG(L12W) | This study |
| pMZ96 | pSTD689 | HA-MBP-YqfG(L14P) | This study |
| pSTD881 | pSTD689 | HA-MBP-RseA140 | *(Akiyama et al, 20*04) |
| pSTD892 | pTYE007 | RseP(Cys-less)-His_6_-Myc | *(Koide et al, 20*07) |
| pSTD1017 | pSTD689 | HA-MBP-RseA(LY1)140 | *(Koide et al, 20*07) |
| pYH18 | pTWV228 | HA-RseA148 | *(Hizukuri and Akiyama, 20*12) |
| pYH19 | pTWV228 | HA-MBP-RseA148 | *(Hizukuri and Akiyama, 20*12) |
| pYH124 | pSTD689 | HA-MBP-RseA(LY1)148 | *(Hizukuri and Akiyama, 20*12) |

Akiyama Y, Kanehara K, Ito K. 2004. RseP (YaeL), an *Escherichia coli* RIP protease, cleaves transmembrane sequences. *EMBO J* **23:** 4434-4442. doi: 10.1038/sj.emboj.7600449

Akiyama Y, Yoshihisa T, Ito K. 1995. FtsH, a membrane-bound ATPase, forms a complex in the cytoplasmic membrane of *Escherichia coli*. *J Biol Chem* **270:** 23485-43490. doi: 10.1074/jbc.270.40.23485

Cherepanov PP, Wackernagel W. 1995. Gene disruption in *Escherichia coli*: Tc^R^ and Km^R^ cassettes with the option of Flp-catalyzed excision of the antibiotic-resistance determinant. *Gene* **158:** 9-14. doi: 10.1016/0378-1119(95)00193-A

Guzman LM, Belin D, Carson MJ, Beckwith J. 1995. Tight regulation, modulation, and high-level expression by vectors containing the arabinose P_BAD_ promoter. *J Bacteriol* **177:** 4121-4130.

Hizukuri Y, Akiyama Y. 2012. PDZ domains of RseP are not essential for sequential cleavage of RseA or stress-induced σ^E^ activation *in vivo*. *Mol Microbiol* **86:** 1232-1245. doi: 10.1111/mmi.12053

Kanehara K, Akiyama Y, Ito K. 2001. Characterization of the *yaeL* gene product and its S2P-protease motifs in *Escherichia coli*. *Gene* **281:** 71-79. doi: 10.1016/S0378-1119(01)00823-X

Kanehara K, Ito K, Akiyama Y. 2003. YaeL proteolysis of RseA is controlled by the PDZ domain of YaeL and a Gln-rich region of RseA. *EMBO J* **22:** 6389-6398. doi: 10.1093/emboj/cdg602

Kihara A, Akiyama Y, Ito K. 2001. Revisiting the lysogenization control of bacteriophage lambda. Identification and characterization of a new host component, HflD. *J Biol Chem* **276:** 13695-13700. doi: 10.1074/jbc.M011699200

Koide K, Ito K, Akiyama Y. 2008. Substrate recognition and binding by RseP, an *Escherichia coli* intramembrane protease. *J Biol Chem* **283:** 9562-9570. doi: 10.1074/jbc.M709984200

Koide K, Maegawa S, Ito K, Akiyama Y. 2007. Environment of the active site region of RseP, an *Escherichia coli* regulated intramembrane proteolysis Protease, assessed by site-directed Cysteine alkylation. *J Biol Chem* **282:** 4553-4560. doi: 10.1074/jbc.M607339200

Young TS, Ahmad I, Yin JA, Schultz PG. 2010. An enhanced system for unnatural amino acid mutagenesis in *E. coli*. *J Mol Biol* **395:** 361-374. doi: 10.1016/j.jmb.2009.10.030
